# Diffuse Large B-Cell Lymphoma Presenting With Superior Vena Cava Syndrome and Recurrent Pleural Effusions in a Patient With Angelman Syndrome: A Case Report

**DOI:** 10.7759/cureus.110668

**Published:** 2026-06-11

**Authors:** Aariez Khalid, Szymon Matejuk, Ada Chaeli Van Der Zijp-Tan, Nathaniel Van Horn, Katrina Jiang, Brian Fouty

**Affiliations:** 1 Internal Medicine, University of South Alabama, Mobile, USA; 2 Radiology, University of South Alabama, Mobile, USA; 3 Family Medicine, University of South Alabama, Mobile, USA; 4 Pulmonary and Critical Care Medicine, University of South Alabama, Mobile, USA; 5 Pathology and Laboratory Medicine, University of South Alabama, Mobile, USA; 6 Pulmonary and Critical Care, University of South Alabama, Mobile, USA

**Keywords:** angelman syndrome, bilateral pleural effusion, large b-cell lymphoma, mediastinal lymph nodes, pulm-crit

## Abstract

Superior vena cava (SVC) syndrome is an uncommon but important presentation of mediastinal malignancy and can rapidly progress to respiratory compromise. We report a case of diffuse large B-cell lymphoma (DLBCL) in a patient with Angelman syndrome and seizure disorder, highlighting the diagnostic and airway challenges encountered during evaluation.

A 42-year-old woman with Angelman syndrome presented with progressive neck and facial swelling after two weeks of fever, chills, and rhinorrhea. She reported worsening dyspnea and was admitted for concern for superior vena cava syndrome. Chest imaging showed extensive mediastinal, hilar, and cervical lymphadenopathy with bilateral pleural effusions and atelectasis, raising concern for lymphoma or metastatic disease. Cardiothoracic surgery was consulted, but mediastinoscopy was deferred because of her short stature and the location and size of the mediastinal mass. The patient’s caregiver initially refused thoracentesis. Her respiratory status worsened, requiring high-flow oxygen for hypoxemia. She was transferred to a tertiary center for further evaluation.

At the tertiary center, pulmonology deferred endobronchial ultrasound because of concern that adequate lymph node tissue could not be obtained due to the patient’s small airway, which could permit only a 6 mm endotracheal tube. The patient subsequently underwent cervical lymph node core needle biopsy and thoracentesis. Thoracentesis yielded approximately 800 mL of yellow-green pleural fluid that had 32% lymphocytes and was exudative based on lactate dehydrogenase (LDH). Pleural fluid flow cytometry was positive for malignancy, showing an abundant monotonous lymphoid population and kappa-restricted clonal B-cells. Thoracentesis on the contralateral side showed an exudative effusion with similar findings. Pleural fluid flow cytometric immunophenotyping showed kappa clonal B-cells expressing CD19 and CD20, and lacking CD10, CD200, and CD38, with equivocal expression of CD5. Cervical lymph node pathology confirmed diffuse large B-cell lymphoma. The patient was started on rituximab, cyclophosphamide, doxorubicin, vincristine, and prednisone (R-CHOP) with a good clinical response. Her course was complicated by prolonged mechanical ventilation due to respiratory muscle weakness requiring tracheostomy.

This case illustrates an unusual presentation of diffuse large B-cell lymphoma with superior vena cava syndrome and bilateral malignant pleural effusions. Diagnosis was delayed due to the significant procedural barriers as a result of her small stature and microcephaly associated with her Angelman syndrome. Prompt multidisciplinary evaluation and alternative tissue acquisition were essential for diagnosis and treatment.

## Introduction

Diffuse large B-cell lymphoma (DLBCL) is the most common subtype of non-Hodgkin lymphoma and may present with rapidly enlarging lymphadenopathy, extranodal involvement, or symptoms related to mass effect [1]. When the mediastinum is involved, patients can develop superior vena cava (SVC) syndrome, pleural effusions, and progressive dyspnea, which require prompt recognition and treatment [2]. Establishing a tissue diagnosis is essential, but this can be challenging when the lesion is anatomically difficult to access or when airway concerns limit standard bronchoscopic or surgical approaches. Angelman syndrome is a rare neurogenetic condition characterized by developmental delay, speech impairment, seizures, and distinctive physical findings. Reports of lymphoma in patients with Angelman syndrome are limited, and the coexistence of this disorder with an aggressive thoracic malignancy adds further diagnostic complexity. Short stature, neuromuscular features, and procedural risk may also restrict conventional biopsy options, making multidisciplinary planning especially important [3]. We present a case of diffuse large B-cell lymphoma presenting with extensive mediastinal lymphadenopathy and suspected superior vena cava syndrome in a patient with Angelman syndrome, highlighting the diagnostic and procedural challenges encountered during evaluation.

## Case presentation

**Figure 1:**
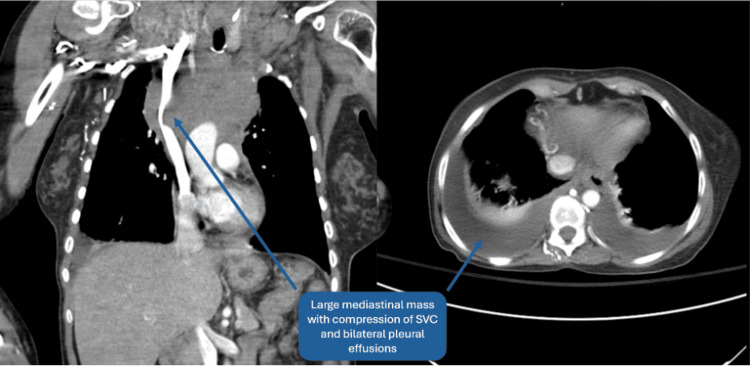
Chest CT with contrast: coronal series demonstrates a large mediastinal mass with compression of the superior vena cava, and axial series demonstrates moderate bilateral pleural effusions. CT: computed tomography

A 42-year-old woman with a history of Angelman syndrome and seizure disorder presented with progressive neck and facial swelling following two weeks of fever, chills, and rhinorrhea that had initially been treated as an upper respiratory infection. She subsequently developed worsening dyspnea and was admitted for concern for superior vena cava (SVC) syndrome. Initial chest imaging demonstrated extensive mediastinal, hilar, and cervical lymphadenopathy concerning for lymphoma or metastatic disease, along with mild bilateral pleural effusions and associated atelectasis. Computed tomography (CT) of the chest with contrast, including coronal and axial series, revealed a large mediastinal mass with compression of the SVC and moderate bilateral pleural effusions (Figure 1).

**Figure 2:**
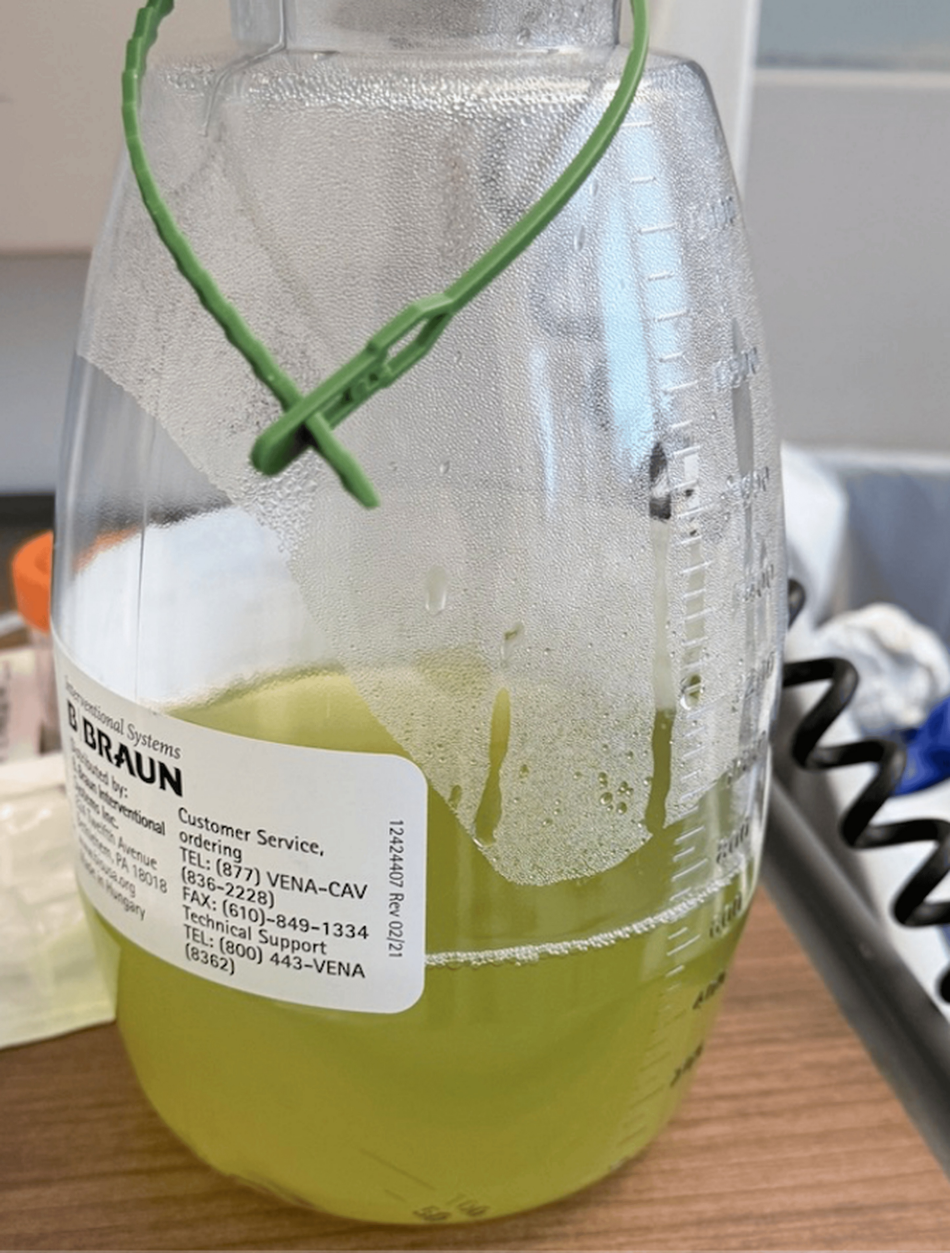
Gross appearance of pleural fluid obtained via thoracentesis, yielding approximately 800 mL of green-tinged fluid.

Cardiothoracic surgery was consulted for mediastinoscopy; however, she was deemed a poor surgical candidate due to her short stature and the size and location of the mediastinal mass. Pulmonology deferred endobronchial ultrasound due to concern for a difficult airway related to her short stature, with inability to obtain sufficient lymph node cores to make a diagnosis. Peripheral blood cytology was unremarkable, as it indicated no abnormal hematolymphoid populations and no circulating blasts. After initially refusing, the patient’s caregiver eventually agreed to a thoracentesis, which removed approximately 800 mL of green-tinged pleural fluid from the right pleural space (Figure 2).

The pleural fluid was exudative with 32% lymphocytes. Pleural fluid cytology revealed evidence of a B-cell lymphoma, and flow cytometry identified a kappa-restricted clonal B-cell population expressing CD19 and CD20, and weak CD5, consistent with a B-cell lymphoproliferative disorder, with differential considerations including mantle cell lymphoma or small lymphocytic lymphoma. Thoracentesis done on the left side (for relief of respiratory symptoms) was also consistent with a malignant effusion.

**Figure 3:**
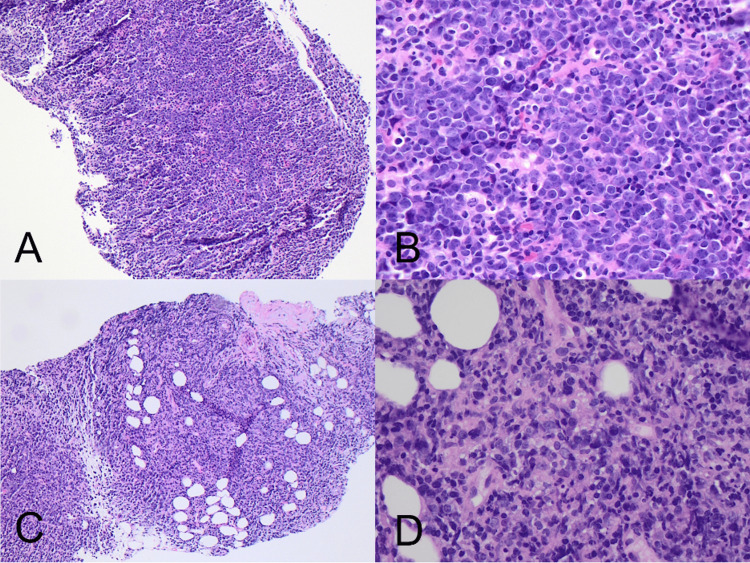
A and B: Representative core biopsy showing effacement of lymphoid architecture by sheets of large, high-grade neoplastic cells, consistent with DLBCL (A: H&E, 10×; B: H&E, 40×). C and D: Separate representative core demonstrating similar morphologic features (C: H&E, 10×; D: H&E, 40×). DLBCL: diffuse large B-cell lymphoma, H&E: hematoxylin and eosin

A cervical lymph node core needle biopsy was then performed. Histopathologic evaluation of the cervical lymph node biopsy demonstrated effacement of lymphoid architecture by sheets of large, high-grade neoplastic cells without an identifiable low-grade component. Immunohistochemical staining, performed with appropriate positive and negative controls, showed CD3 and CD5 highlighting background T cells, while CD20 highlighted neoplastic B cells in a diffuse growth pattern. The neoplastic cells were negative for CD10 and cyclin D1, and positive for MUM1, BCL-2, and BCL-6. The Ki-67 proliferation index was approximately 90%, and CD21 demonstrated the absence of a dendritic cell meshwork, supporting a high-grade process. Burkitt lymphoma was considered unlikely given the absence of CD10 expression and the presence of BCL-2 expression. Overall, the morphologic and immunophenotypic findings supported a diagnosis of DLBCL (Figure 3).

Representative core biopsy sections showed effacement of lymphoid architecture by sheets of large neoplastic cells at both low- and high-power magnification, with similar morphologic features observed across separate cores.

Final pathology confirmed DLBCL. The patient was initiated on rituximab, cyclophosphamide, doxorubicin, vincristine, and prednisone (R-CHOP) therapy, with regression of her thoracic adenopathy and decreased obstruction of the superior vena cava. Her hospital course was complicated by prolonged mechanical ventilation requiring tracheostomy due to respiratory weakness, likely related to a combination of intensive care unit (ICU)-acquired myopathy and steroid-induced myopathy associated with chemotherapy. She was ultimately discharged to a long-term acute care hospital (LTACH) with outpatient follow-up for continuation of chemotherapy.

## Discussion

This case highlights several important diagnostic and management challenges in a patient with an atypical presentation of aggressive lymphoma complicated by suspected superior vena cava (SVC) syndrome. The presence of developmental disability from Angelman syndrome added further complexity, limiting procedural options and delaying tissue diagnosis.

Diffuse large B-cell lymphoma (DLBCL), the most common subtype of non-Hodgkin lymphoma, is an aggressive malignancy that often presents with rapidly enlarging lymphadenopathy and systemic “B symptoms” such as fever and weight loss. Mediastinal involvement, as seen in this patient, can lead to compression of adjacent structures, including the SVC, resulting in SVC syndrome. Malignancy accounts for approximately 60%-85% of SVC syndrome cases, with lymphoma representing a significant proportion alongside lung cancer [3,4]. Early recognition is critical, as progression can lead to respiratory compromise, cerebral edema, and hemodynamic instability.

In this case, the patient initially presented with symptoms suggestive of an upper respiratory infection, which delayed further evaluation. This reflects a known challenge in lymphoma diagnosis, where early symptoms may be nonspecific. The progression to facial and neck swelling, along with dyspnea, appropriately raised concern for SVC syndrome and prompted imaging that revealed extensive lymphadenopathy. Cross-sectional imaging remains the cornerstone of initial evaluation, providing both diagnostic and staging information [5]. In particular, contrast-enhanced CT allows for rapid assessment of vascular compression, collateral formation, and extent of disease, all of which are essential in guiding the urgency of intervention [6].

Obtaining tissue diagnosis in this patient was challenging. Mediastinoscopy, often considered the gold standard for mediastinal lymph node sampling, was not feasible due to the anatomical constraints of a small neck. Similarly, endobronchial ultrasound-guided transbronchial needle aspiration (EBUS-TBNA), a less invasive alternative with a sensitivity exceeding 85% for diagnosing mediastinal lymphadenopathy, was deferred because her small airway limited the size of her endotracheal tube to 6 mm, which would have made it difficult to obtain sufficient biopsies [7]. Ultimately, the combination of pleural fluid flow cytometry and cytology and cervical lymph node biopsy led to the diagnosis.

The diagnostic utility of pleural fluid flow cytometry in this case is notable. Thoracentesis was initially delayed because the patient’s caregiver refused to give consent, but once obtained, it was critical in providing an answer. Flow cytometry can detect monoclonal B-cell populations and immunophenotypic markers such as CD19, CD20, and CD5, helping narrow the differential diagnosis. However, distinguishing between entities such as mantle cell lymphoma and small lymphocytic lymphoma often requires tissue biopsy and additional studies, including immunohistochemistry and cytogenetics [8]. The incorporation of fluorescence in situ hybridization (FISH) and molecular studies further refines classification, particularly in identifying high-risk subtypes such as “double-hit” lymphomas involving MYC and BCL2 or BCL6 rearrangements [9]. Ultimately, excisional lymph node biopsy remains the gold standard for lymphoma diagnosis and classification, as reflected in this patient’s final diagnosis of DLBCL.

An additional complexity in this case was the patient’s underlying Angelman syndrome, a neurogenetic disorder characterized by developmental delay, limited verbal communication, and behavioral differences. These factors can complicate symptom reporting, physical examination, and procedural planning. Airway management concerns in patients with craniofacial or anatomical variations may limit the use of standard diagnostic techniques, as seen here. This underscores the importance of individualized, multidisciplinary care involving pulmonology, thoracic surgery, oncology, and critical care teams.

Once the diagnosis of DLBCL was established, the patient was initiated on R-CHOP therapy (rituximab, cyclophosphamide, doxorubicin, vincristine, and prednisone), the standard first-line treatment. R-CHOP has significantly improved survival outcomes in DLBCL, with cure rates approaching 60%-70% depending on risk stratification [10]. Risk stratification tools such as the International Prognostic Index (IPI), which incorporates age, stage, performance status, extranodal involvement, and lactate dehydrogenase (LDH) levels, remain central to prognostication and treatment planning [11]. In select high-risk patients, dose-adjusted regimens or incorporation of novel agents may be considered.

Supportive care also plays a critical role in management. Patients with bulky mediastinal disease are at risk for tumor lysis syndrome, airway compromise, and rapid clinical deterioration following initiation of therapy. Prophylactic measures, including aggressive hydration and monitoring of electrolytes, are essential, particularly in patients with high tumor burden [12]. Additionally, corticosteroids are sometimes used empirically in suspected lymphoma with compressive symptoms; however, their use prior to biopsy can obscure histopathologic diagnosis and should be approached cautiously when feasible.

Despite appropriate treatment, the patient’s course was complicated by prolonged respiratory failure requiring mechanical ventilation and eventual tracheostomy. This likely reflects a combination of factors, including mass effect from bulky mediastinal disease, pleural effusions, and overall critical illness. Patients with SVC syndrome and mediastinal masses are at increased risk for airway compromise, particularly during sedation or invasive procedures. Careful planning and close monitoring in an intensive care setting are essential in such cases [13]. Endovascular stenting has emerged as an option for rapid symptomatic relief in severe SVC syndrome, although it is typically reserved for refractory or life-threatening cases and used in conjunction with definitive oncologic therapy [14].

This case also illustrates the importance of flexibility in diagnostic strategy. When standard approaches such as mediastinoscopy or EBUS are not feasible, alternative methods, including peripheral lymph node biopsy and fluid analysis, can provide critical diagnostic information. Early involvement of tertiary care centers may facilitate access to specialized expertise and resources, improving outcomes.

In summary, this case emphasizes the need for high clinical suspicion for malignancy in patients with progressive lymphadenopathy and respiratory symptoms, even when initial presentations suggest benign etiologies. It highlights the challenges of obtaining tissue diagnosis in anatomically complex patients and the value of multidisciplinary care. Finally, it reinforces the importance of the timely initiation of therapy in aggressive lymphomas such as DLBCL to achieve optimal outcomes.

## Conclusions

This case highlights the diagnostic and procedural challenges of evaluating aggressive mediastinal lymphoma in a patient with Angelman syndrome and complex anatomical considerations. Limitations in standard biopsy approaches required alternative diagnostic strategies, ultimately leading to the diagnosis of diffuse large B-cell lymphoma through cervical lymph node biopsy and pleural fluid analysis. The case emphasizes the importance of maintaining suspicion for malignancy in patients with progressive lymphadenopathy and respiratory compromise, as well as the value of multidisciplinary management and flexibility in diagnostic planning when conventional procedures are not feasible.
